# Influence of neurological severity and degree of dependence of patients after stroke on nursing workload: a retrospective cohort

**DOI:** 10.1590/1980-220X-REEUSP-2024-0302en

**Published:** 2025-07-14

**Authors:** Felipe da Cruz Lima, Joathan Borges Ribeiro, Samia Denadai, Lilia Souza Nogueira, Renata Eloah de Lucena Ferretti-Rebustini

**Affiliations:** 1 Universidade de São Paulo, Escola de Enfermagem, São Paulo, SP, Brazil.; 2 Hospital Sírio-Libanês, Unidade de Terapia Intensiva, São Paulo, SP, Brazil.; 3 Universidade de São Paulo, Escola de Enfermagem, Enfermagem Médico-Cirúrgica, São Paulo, SP, Brazil.

**Keywords:** Stroke, Nursing, Functional Status, Workload

## Abstract

**Objective:**

To verify the influence of neurological severity and the degree of dependence of patients after stroke on the nursing workload required during hospitalization in the Intensive Care Unit (ICU).

**Method:**

Retrospective cohort of post-stroke patients admitted between 2019-2022 to a private ICU in the city of São Paulo, Brazil. Neurological severity was measured through the *National Institutes of Health Stroke Scale (NIHSS)*, the level of dependency through the Katz Index, and the nursing workload through the *Nursing Activities Score* (NAS). Information from the 98 patients was collected from electronic medical records and the mixed effects model was applied in the analyses.

**Results:**

In a sample of older people (76.1 ± 14.8 years) and consisting predominantly of men (62.4%), it was observed that neurological severity (p < 0.001) and the degree of dependence (p = 0.002) independently predict NAS values, without any relation to the time at which this variable was measured (admission, average day of hospitalization, or discharge from the ICU).

**Conclusion:**

Neurological severity and degree of dependence influence the nursing workload required by post-stroke patients in the ICU and should be considered when determining the size of the team.

## INTRODUCTION

Annually, cerebrovascular events are responsible for approximately seven million deaths worldwide, being the second cause of death in developing countries^(1)^. In Brazil, stroke, one of the most frequent cerebrovascular events, is the main cause of disability in the population over 50 years of age and represents 32.6% of deaths from vascular diseases^(2)^.

An individual affected by a stroke undergoes a series of changes in their metabolism, and it is very important, even in the acute phase of the disease, to measure the severity of the event, predicting and avoiding possible sequelae^(3)^. For this purpose, there are scales that predict neurological severity, with the National Institutes of Health Stroke Scale (NIHSS) being highlighted, which uses the patient’s age and neurological impairment as parameters for calculation^(4)^.

The NIHSS score can range from 0 to 42 points, with scores below 8 being associated with mild and transient neurological deficits, between 8 and 17 corresponding to a moderate event, and scores greater than or equal to 18 corresponding to severe neurological deficit^(4)^. One of the main consequences of these cerebrovascular events is the potential to generate significant degrees of disability, since approximately 70% of those affected are unable to return to their work activities and 50% have difficulties performing activities of daily living (ADLs)^(5)^.

From this perspective, one of the instruments used in clinical practice to assess basic activities of daily living (BADLs) is the Katz Index, which allows identifying the patient’s degree of dependence or incapacity through the assessment of six basic self-care functions: bathing, dressing, eating, using the bathroom, carrying out transfers, and maintaining control over eliminations, classifying the individual as independent, partially dependent, or totally dependent to perform such actions^(6,7)^.

Patients with severe neurological conditions, such as stroke, are considered neurocritical and commonly require specialized care provided in Intensive Care Units (ICU). In this context, the degree of dependence and severity of these patients during intensive treatment may intensify the need for care provided by the Nursing team. Thus, the importance of the nursing team’s role in providing care to patients with stroke becomes evident, since the services provided by these professionals are essential throughout the pathological process. The performance of this category contributes to minimizing the damage caused by changes in sensory-motor functions during care in the acute phase and in the recovery of the disease, promoting better functional independence, including the ability to perform BADLs and, consequently, providing a better quality of life^(8,9)^.

Thus, many individuals who experience severe neurological deficits after stroke are unable to perform BADLs, as well as manage self-care^9^, and studies demonstrate the importance of nursing interventions during all stages of treatment of these patients to achieve satisfactory outcomes^(8,10,11)^. Studies portray the impact of stroke on the need for nursing care required by patients after hospital discharge^(11,12)^. However, few studies have investigated the use of Nursing Activities Score (NAS) in neurological patients and the results found indicate a direct relationship between the severity of the disease and the nursing workload^(10,13)^. Neurological patients with a higher degree of criticality and those who may progress to death require more bedside care and complex therapies, increasing the nursing team workload^(10)^.

Therefore, the literature broadly demonstrates the applicability of NAS in neurological patients^(10,13)^, regardless of the condition that led them to the ICU, as in the case of stroke. Thus, the results of the present study may be extremely relevant for planning care aimed at the special needs of this group during intensive treatment, in addition to providing essential information for adequately dimensioning the nursing team for safe and effective care.

Therefore, the question is: does the neurological severity (NHISS) of the stroke patient influence the nursing workload during ICU admission? Does the patient’s degree of dependence on performing BADLs impact the request for care?

Therefore, the objective of the study was to verify the influence of neurological severity and the degree of dependence of post-stroke patients on the nursing workload required during ICU hospitalization.

## METHOD

### Design of Study

This is a retrospective cohort that analyzed patients with stroke treated in the emergency room of a private hospital in the city of São Paulo, capital of the state, from January 2019 to December 2022. The study site is a private hospital complex located in the city of São Paulo, Brazil, consisting of approximately 50 active general ICU beds, distributed among 3 wards, one of which is intended for patients with neurological impairment.

### Population

The study population consisted of 198 patients admitted during the study period via emergency care and who had, through neuroimaging, confirmed ischemic or hemorrhagic stroke in the institution’s emergency room and were referred to the neurological ICU.

### Sample Definition

From the list provided by the institutional protocols department regarding the 198 patients admitted to the emergency room, a sample calculation was performed, considering a model with partial effect size η^2^ = 0.05. For this purpose, the repeated measures ANOVA model was used, which is closest to the mixed effects model for sizing samples. With type I and II errors of 5% each, it is concluded that it would be necessary to observe at least 72 subjects. However, to compensate for possible losses, an addition of 30% was planned, which resulted in a final sample estimated at 93 participants. Post-hoc analysis to investigate the achieved power indicated a study power (probability 1 β error) of 0.80, calculated for an effect size of 0.30 in a sample of 93 participants.

The study included individuals admitted to the emergency room in question, from January 2019 to December 2022, with a stroke confirmed by neuroimaging, who were 18 years of age or older and who were admitted to the neurological ICU after initial management. Individuals were excluded from the sample due to the absence of the NIHSS calculated upon admission to the emergency room, stay in the ICU for a period of less than 24 hours, and lack of information recorded in the electronic medical record regarding the variables of interest to the study.

### Study Variables

The independent variables of the study were neurological severity according to the NIHSS^4^ assigned and the degree of dependence of patients on BADLs, according to the Katz Index^7^. The NIHSS uses as parameters the individual’s age, level of consciousness, language, visual field loss, extraocular movements, motor strength, ataxia, dysarthria, and sensory loss. Its score ranges from 0 to 42 points, where higher scores are related to lower chances of positive results post-event^(6,7)^.

The Katz Index was used in its categorical form, assigning a score to each item as follows: 0 - for participants who are totally dependent (dependent); 0.5 patients who require partial assistance (partially dependent) and 1 - patients who perform activities without assistance (independent). The resulting final score was between 0-6 points^(7)^.

The nursing workload, a dependent variable, was identified by NAS, whose final score (maximum of 176.8%) reflects the percentage of time, during 24 hours, that the patient required assistance from the nursing professional, through the analysis of 23 nursing interventions^(14)^.

As secondary variables, patient characteristics (sex, age, skin color, marital status, religion, level of education, body mass index – BMI, Charlson comorbidity index), type of stroke (ischemic or hemorrhagic), support used in intensive care, indicators of physiological severity, such as Sequential Organ Failure Assessment (SOFA) and Simplified Acute Physiology Score III (SAPS 3) upon admission to the ICU, pre-stroke Rankin scale, as well as clinical data such as length of stay in the ICU and in the hospital, and hospital outcome (survivor or non-survivor) were evaluated.

### Data Collection

This study was carried out by collecting data from patients’ electronic medical records and the Epimed Solutions® system between August and September 2023. Additional information was provided by the institutional protocols group (Clinical Outcome), a sector that monitors the activation and execution of care protocols, such as in stroke, and records each patient’s outcome.

Through the application of a data collection instrument developed by the researchers, stroke calls made via emergency care were extracted and, from these, information was analyzed on patients who had a confirmed diagnosis with referral to the ICU, in addition to the NIHSS admission score performed by the neurologist on duty, in accordance with the institutional protocol. Furthermore, the NIHSS scored items were collected to identify the deficits present in each patient at the time of admission to the emergency room.

To record the level of dependency for BADLs, the Katz Index was used at three moments: status prior to the ICU through information provided by family members/caregivers, admission to the ICU, and upon discharge from the unit. Completion of the Katz index (admission and discharge from the ICU) was based on the nursing team’s records, which were related to the daily care provided to the group in question. Furthermore, with the aim of completing this information, we sought to associate it with the assessments of the physiotherapy team that applies the Katz index daily during the functional assessment of patients and is trained in the applicability of the index during the institutional hiring period.

The NAS, collected from the electronic medical record, was calculated by the ICU nurses in question, daily, at 5 pm. Thus, for this study, NAS was recorded at three moments during the patient’s stay in the ICU: admission, intermediate (average day of hospitalization), and upon discharge from the unit.

Finally, to ensure the reliability of the extracted data and to reduce selection and data reporting biases, these were collected by a single researcher, using a standardized collection instrument. Furthermore, medical records that did not contain all the reported variables had their service numbers, used to locate them, excluded from the database and, consequently, from the study.

### Data Analysis and Treatment

Data were analyzed using the statistical package R version 4.3.0. Continuous variables were presented as mean, standard deviation, median and quartiles, and categorical variables as absolute (n) and relative (%) frequency. To test the influence of independent variables (neurological severity and level of dependence) on nursing workload, the mixed effects model was used, employing the Wald type III Chi-square test. This methodology allowed the evaluation of a patient’s measurements throughout the studied period, as well as their variation. The significance level adopted was 0.05, in two-tailed tests, considering an α of 5%.

### Ethical Aspects

This study followed the ethical principles that regulate research involving human beings in accordance with Resolutions No. 466/12 and 510/16 of the National Health Council. As recommended by the institution, the project was initially authorized by the coordinators of the units where the research would be conducted and submitted to the Centro de apoio ao pesquisador (*AVAP*), research management platform of the study hospital. Afterwards, the project was submitted to the Research Ethics Committee (*CEP*), with subsequent approval (Opinion No. 6.176.270 and CAAE No. 70539323.0.0000.5461). The *CEP* authorized the waiver of the application of the Free and Informed Consent Form to patients, since data collection was performed secondarily through the Epimed Solutions® system and electronic medical records.

## RESULTS

When analyzing the profile of the patients included in the present study, we identified a predominance of males (n = 58; 62.4%), white skin color (n = 88; 94.6%), married marital status (n = 60; 64.5%), finished higher education (n = 70; 75.3%), and Catholic religion (n = 58; 62.3%). The mean age was 76.1 ± 14.8 years, median 81 years and mean Body Mass Index (BMI) 27.4 ± 4.7.

There was a predominance of patients with stroke of ischemic nature (95.7%), admitted to the ICU due to clinical causes (94.6%), coming from the emergency room (82.8%), independent based on clinical evaluation (79.6%), and with no disabling symptoms (46.2%) according to the pre-stroke Rankin scale.

Regarding the support used in the ICU, 12.9% of the sample required mechanical ventilation and 4.3% progressed to tracheostomy. The use of vasopressors was recorded in 17.2% of the sample, only 2.1% required renal replacement therapy, and there was no use of extracorporeal membrane oxygenation.

The mean and median NHISS scores at admission to the emergency room were 8.5 ± 6.2 and 7, respectively, and a higher frequency of deficits related to central facial paralysis (29%), sensitivity (26.9% with mild to moderate loss and 12.9% with severe loss) and speech ability, evidenced by dysarthria (25.8% with mild to moderate dysarthria and 23.7% with severe dysarthria) and better language (16.1% with severe aphasia) were found. The other findings related to the comorbidity index, severity and length of stay in the ICU and hospital are shown in Table 1.

**Table 1:** Characterization of the sample according to Charlson comorbidity index, severity and length of hospital stay – São Paulo, SP, Brazil, 2023.

	Mean	SD	Min	Q1	Median	Q3	Max	Min. CI	Max. CI
NIHSS	8.5	6.2	1.0	3.0	7.0	14	24	7.3	9.8
Charlson	1.4	1.6	0.0	0.0	1.0	2.0	6.0	1.2	1.8
SAPS 3	51.3	8.3	34.0	46.0	54.0	56.0	82.0	49.6	53.0
SOFA	2.2	2.6	0.0	0.0	2.0	3.0	13.0	1.8	2.8
Length of stay in the ICU	5.6	6.0	1.0	2.0	3.0	7.0	40.0	4.6	7.2
Length of hospital stay	19.5	19.1	2.0	8.0	12.0	23.0	107.0	16.2	24.1
Hospital									
Katz Index									
Prior	5.3	1.1	1.5	5.0	6.0	6.0	6.0	5.0	5.5
Admission	3.1	1.8	0.5	1.0	3.0	4.5	6.0	2.7	3.4
Discharge	3.2	2.1	0.0	2.0	3.0	4.5	6.0	2.8	3.6
NAS									
Admission	50.8	20.3	24.8	42.7	49.4	71.3	108.7	54.1	62.3
Intermediate	56.5	17.3	31	42.3	51.6	67.8	101.1	53.2	60.2
Discharge	51.7	14.7	26.6	40.8	49.3	61.5	88.5	48.9	54.8

Min – Minimum; Max – Maximum; Q1 – Quartile 1; Q3 – Quartile 3; CI – Confidence Interval; NIHSS – *National Institute of Health Stroke Scale*; Charlson – *Charlson Comorbidity Index*; SAPS3 – *Simplified Acute Physiology Score 3;* SOFA – *Sequential Sepsis*-*related Organ Failure Assessment*; ICU – Intensive Care Unit; NAS – *Nursing Activities Score*; Katz – *Katz Index*.

Regarding the Katz Index collected from family members, patients had an average value of 5.3 in the previous state, reflecting independence in the group. After the cerebrovascular event, the mean value reduced significantly to 3.1, with a slight increase at the time of discharge from the ICU to 3.2.

Regarding NAS, a reduction was observed between measurement moments during the ICU stay. At the time of admission to the critical unit, higher NAS values were observed, with a mean of 58.0% and a median of 49.4%; in the intermediate collection, a slight change in the scores was noted, 56.5% and 51.6%, while at discharge, the reduction in NAS was maintained, 51.7% and 49.3%, respectively.

Regarding the categorization of dependence based on the Katz Index, it was observed that the level of dependence increases in all items analyzed at the time of admission to the ICU compared to the patient’s behavior before the cerebrovascular event (previous state). However, there was an improvement in the percentages at the time of discharge, with the exception of the item “transfer”, which showed an exponential increase in dependency from 1.1% at admission to 30.1% at discharge from the ICU.

**Figure 1:**
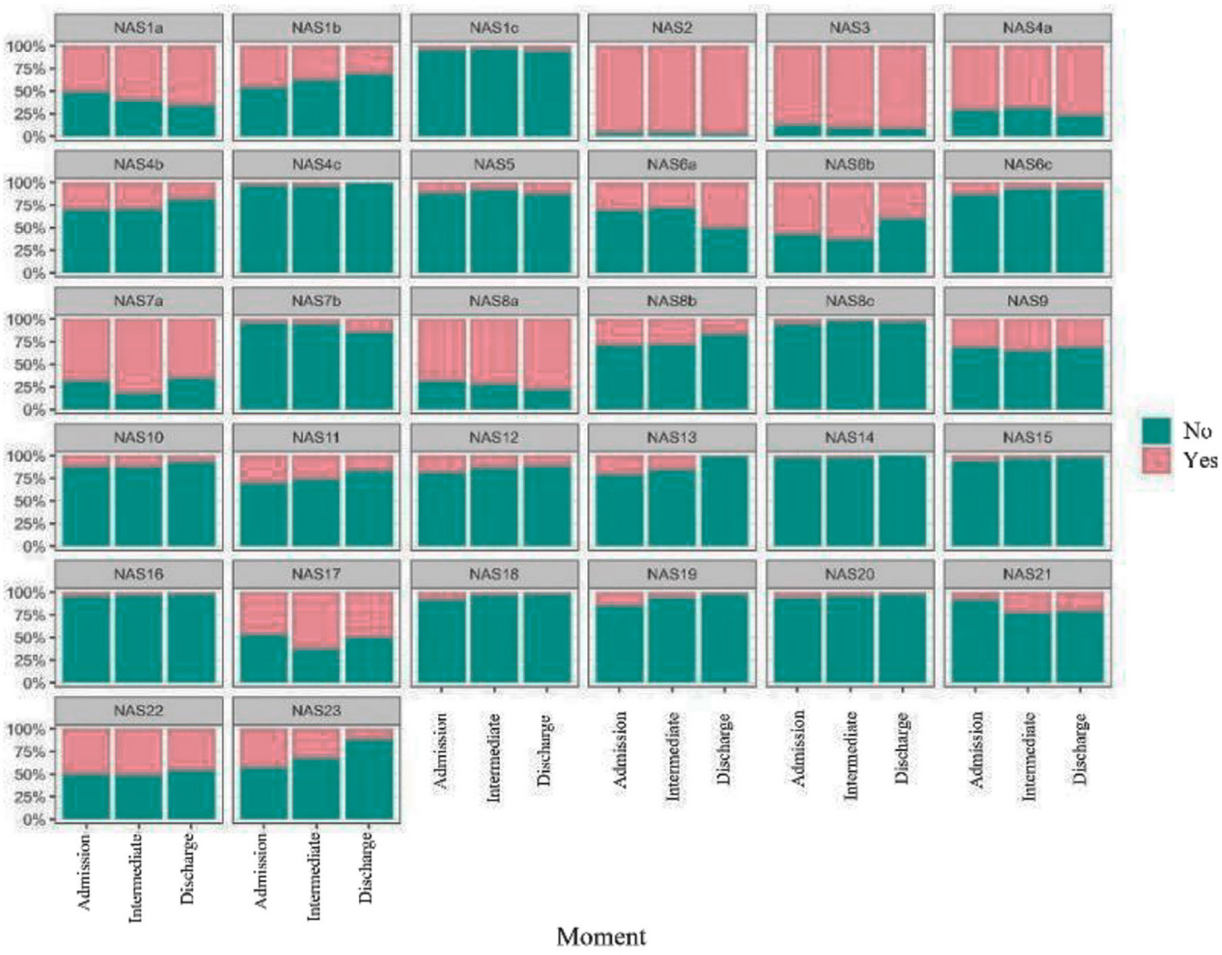
Frequency of NAS items scored at different measurement times.

Regarding the NAS items, a significant frequency of interventions related to basic care was observed, such as in item 4a (performance of hygiene procedures), with 71% upon admission to the ICU, followed by 68.8% in the intermediate stage and, finally, 76.3% upon discharge. The same applied to item 6b, need for mobilization and positioning more than 3 times in 24 hours or with two nursing professionals, scoring 57.7%, 62.4% and 39.8%, respectively for the moments mentioned. Other interventions with more expressive findings were related to monitoring and controls (item 1a); laboratory, biochemical, and microbiological investigations (item 2); administration of medications, except vasoactive drugs (item 3), support and care for family members (item 7a), routine administrative and managerial tasks (item 8a) and quantitative measurement of urinary output (item 17). Figure 1 shows the NAS item scores at the three time points measured in the ICU.

Table 2 shows the relationship between the NHISS score at the time of emergency room admission and the demand for nursing care measured by the NAS at the three time points (admission, intermediate, and discharge). It is noted that each NIHSS unit increases approximately 1.5 points in the NAS at admission, 1.3 on the average day of hospitalization (intermediate), and 0.7 at discharge from the ICU (p = 0.013).

**Table 2:** NHISS scores and NAS values at different measurement times in the ICU – São Paulo, SP, Brazil, 2023.

	Moment (NAS)	Coefficient	SD	Min. CI	Max CI	p Value
NHISS	Admission	1.589	0.267	1.061	2.116	0.013
	Intermediate	1.335	0.267	0.808	1.863	
	Discharge	0.767	0.267	0.240	1.294	

SD – Standard deviation; Min CI – Minimum confidence interval; Max CI – Maximum confidence interval.

**Figure 2:**
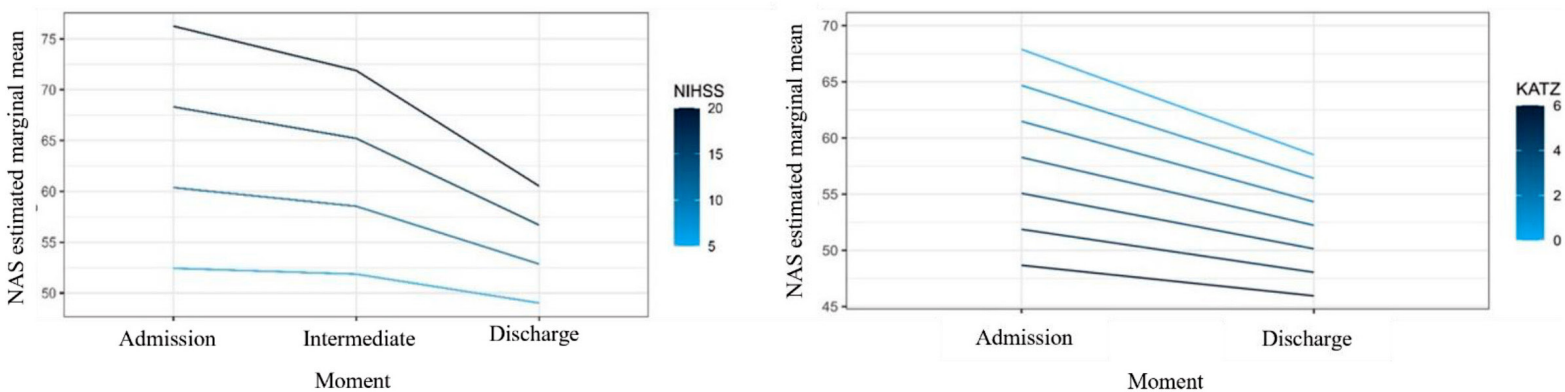
Graphs of the relationship between the NAS score and the NIHSS and Katz Index at different measurement times in the ICU.

Figure 2 shows that the higher the NIHSS value, the higher the estimated marginal mean in the NAS, regardless of the moment analyzed. Furthermore, it is observed how much the demand for care required by the patient changes in relation to their NHISS score upon admission and that patients with higher NHISS progressed with a significantly (p = 0.013) more pronounced drop in the NAS value during hospitalization, unlike those admitted to the ICU with lower NHISS values, who present less variation in the recorded NAS scores. Similar behavior was also observed between the Katz Index and the NAS of the patients analyzed at the three moments of ICU admission (p = 0.328).

Table 3 presents the analysis of the influence of the NHISS and the Katz Index on the nursing workload through the NAS measured at the three moments. It is noted that the NIHSS (p < 0.001) and the Katz Index (p = 0.002) independently predict NAS values and there is no relationship with the moment at which nursing workload is measured. Thus, patients who are more dependent on BADLs (lower Katz Index values) and those with greater neurological severity observed by the NIHSS will have higher NAS scores, regardless of the measurement time (admission, intermediate, or discharge).

**Table 3:** Influence of the Katz Index and NHISS on the workload measured by the NAS in the ICU at different times – São Paulo, SP, Brazil, 2023.

	X^2^	p-Value (>X^2^)
(Intercept)	218.862	<0.001
Measurement time	0.385	0.535
NIHSS	13.615	<0.001
Katz	10.002	0.002
Moment:NIHSS	3.366	0.067
Moment:Katz	0.957	0.328

X^2^ – Pearson’s chi-square; NIHSS – *National Institute of Health Stroke Scale*; Katz – *Katz index.*

Finally, most patients were discharged from the ICU (94.6%) and survived (91.4%) during their hospital stay.

## DISCUSSION

The present study showed that the NIHSS and the Katz index influence the NAS values independently, without any relation to the moment of measurement of the nursing workload in the ICU (admission, intermediate, or discharge).

The sample was predominantly composed of men and older people. In this sense, it is known that chronic diseases associated with factors such as advanced age and ICU admission negatively affect the functional independence of individuals, so that, as the incidence of diseases increases, the risk of incapacity in basic life activities increases^(15)^. Therefore, it is worth highlighting that cerebrovascular events are directly associated with the degree of dependence of older people^(16)^. This justifies the fact that patients present, prior to the stroke, a greater degree of functional independence with a subsequent decrease in the Katz index score, becoming more dependent after the cerebrovascular event.

In addition, the set of neurological deficits presented by patients as a result of vascular changes is closely related to their severity. Results present in the literature demonstrated the existence of a relationship between mild stroke symptoms, indicated by the NHISS scale, and the expansion or area of neurological injury^(16)^. Considering that scores between 8 and 17 points characterize moderate neurological severity, the present study showed an average NHISS score of 8.5 associated with a higher frequency of deficits related to central facial paralysis, sensitivity and speech ability, as indicated by the rates of dysarthria and better language, in addition to changes in motor strength.

Changes related to language, neglect, visual deficits, motor and sensory activities, generally present in cases considered severe, are often related to less favorable outcomes and, consequently, a greater degree of dependence after the neurological event^(17)^. Thus, the presence of such deficits in the sample in question may be related to changes in the degrees of dependence for performing BADLs.

Furthermore, it is known that the main reason for elderly people being admitted to the ICU is the worsening of chronic diseases, mainly of vascular origin, as occurs in stroke^(17,18)^. Nursing care provided to this population is associated with increased nursing workload^(19)^, as evidenced by mean NAS scores at ICU admission (58.0%), at the intermediate moment (56.5%), and at discharge (51.7%). Similar findings were present in a study developed by Lucchini et al.^(19)^, which recorded an average NAS of 59.33 in ICUs in Italy, as well as in a Brazilian study that analyzed older people who required intensive care^(20)^.

In view of this, as a consequence of physiological changes caused by the aging process itself, often associated with certain degrees of fragility, this public becomes more vulnerable, a fact that is often exacerbated by the pathological conditions that surround them, such as, for example, disabilities caused by stroke^(21,22)^. Regarding the group in question, a predominance of care related to basic activities was noticeable, such as mobilization/positioning and hygiene procedures, including bed baths, present throughout the stay in the ICU. Generally, activities involving hygiene and mobilization are associated with a progressive loss of autonomy to perform basic and instrumental activities, which are accentuated by the varying degrees of deficits caused by the stroke^(8)^.

Other interventions with greater frequency at all times during hospitalization were related to the need for monitoring and to vital signs (item 1), microbiological investigation (item 2), medication, except vasoactive drugs (item 3), and support and care for family members (item 7). These findings are justified once again by the predominant age range in the group, which requires greater continuous monitoring, as a result of the changes undergone by the body, which are mainly affected by health conditions that directly impact the use of continuous medications, aiming to control such conditions, and generally resulting in the so-called polypharmacy^(23,24)^.

Given the psychometric properties of the Katz Index and its versions, it was identified that it is a valid and reliable instrument for determining and evaluating the ability to perform BADLs and mortality of post-stroke patients^(8,25)^. Therefore, the study in question demonstrated a relationship between neurological severity and the ability to perform BADLs, when compared to the previous functional level. With an interaction represented by negative coefficients, it was observed that after the cerebrovascular event there was an influence of the stroke on the functional capacity in the following proportion: 0.299 of the admission Katz and 0.332 of the outcome were reduced in relation to the previous capacity, increasing the level of dependence of the sample.

In addition, evidence points to the direct influence of stroke as a disabling agent for an individual’s functionality and physical independence, demonstrating a direct and acceptable relationship with the Katz index, considered an excellent predictor of post-event disabilities, especially when associating older ages with greater degrees of disability^(26)^. In light of this, the study in question shows a direct interaction between the occurrence of a stroke and the change in the functional capacity. Since interactions between the severity of the disease associated with the nursing workload are common, it is known that the NHISS scale acts as an indicator of neurological severity, generally related to the extent and severity of the cerebrovascular event. The present study showed that patients who presented greater severity initially had higher care demand scores, as well as a more pronounced drop in this index during the ICU admission process. Findings in the literature demonstrated the existence of this relationship in a similar way, by evidencing in a neurological ICU the presence of higher NAS scores associated with patients who presented greater severity according to SAPSII^(13)^.

Thus, it is known that the severity of the disease influences the care provided to the patient and that, in addition to this, their physical dependence will affect the performance of certain activities. However, the severity of the disease should not be the only factor to be considered when determining the nursing care provided, since, given the plurality of patients, other factors, such as functional dependence and level of consciousness, must be considered^(8,19,27)^. In view of this, interaction was then evidenced, independently, between the variables mentioned (NHISS and Katz Index) with the NAS, leading to the inference that both neurological severity and the degree of incapacity will influence the demand for nursing care required by patients in the ICU, regardless of the time of measurement. However, the study has the following limitations: the presence of a sample belonging to a single health institution, with a population with access to supplementary health and greater long-term clinical monitoring, which may limit its external validity. Furthermore, the absence of a segregated analysis of the group studied by type of event (ischemic or hemorrhagic) stands out, with the data being presented in a unified manner.

In contrast, the innovation of this research stands out, since there are no studies in this line, that is, that point out the relationship between the neurological disorder addressed and the demand for nursing care in intensive care. In addition, the good structure of the design established for data analysis stands out, which brings reliability to the findings present in this study.

Finally, considering that nursing workload directly interferes with the quality of care^(28)^, the results presented here have great potential to contribute to clinical practice and research, since they highlight, in an unprecedented way, the intensive nursing care provided to patients with a clinical diagnosis of stroke. Thus, evidence regarding the workload required by post-stroke patients admitted to the ICU contributes to strengthening health management by identifying aspects that can assist in dimensioning the nursing team and daily distribution of patient(s) per professional based on neurological severity, degree of dependence, and other conditions relevant to ensuring the safety of the intensive care provided.

Although this study was conducted with a sample size higher than that estimated by statistical calculation, it is believed that carrying out new investigations on NAS in patients with stroke, especially multicenter ones, and in other health contexts, such as public institutions and non-critical units, can strengthen and support the care practice for this population through the consolidation of more scientific evidence.

## CONCLUSION

Given the above, it is concluded that the degree of neurological severity presented by the NHISS score and functional dependence according to the Katz Index presented by post-stroke patients significantly influence the nursing workload measured by the NAS, regardless of the time of its measurement during ICU admission.

## Data Availability

The entire dataset supporting the findings of this study is available upon request to the corresponding author, Felipe da Cruz Lima. The dataset is not publicly available because the results that comprise the research involve information that compromises the privacy of the research participants.
