# Supplementary material for: Influence of neurological severity and degree of dependence of patients after stroke on nursing workload: a retrospective cohort
Source: Rev Esc Enferm USP. 2025 Jul 14;59:e20240302. doi: 10.1590/1980-220X-REEUSP-2024-0302en (PMC12274075; doi:10.1590/1980-220X-REEUSP-2024-0302en)
Supplement: Translation

# Translation: Influência da gravidade neurológica e grau de dependência de pacientes após acidente vascular encefálico na carga de trabalho de enfermagem: coorte retrospective

**DOI:** 10.1590/1980-220X-REEUSP-2024-0302pt

**Authors:** Felipe da Cruz Lima, Joathan Borges Ribeiro, Samia Denadai, Lilia Souza Nogueira, Renata Eloah de Lucena Ferretti-Rebustini

**Affiliations:** 1 Universidade de São Paulo, Escola de Enfermagem, São Paulo, SP, Brasil.; 2 Hospital Sírio-Libanês, Unidade de Terapia Intensiva, São Paulo, SP, Brasil.; 3 Universidade de São Paulo, Escola de Enfermagem, Enfermagem Médico-Cirúrgica, São Paulo, SP, Brasil.

**Keywords:** Acidente Vascular Encefálico, Enfermagem, Estado Funcional, Carga de Trabalho

## Abstract

**Objetivo:**

Verificar a influência da gravidade neurológica e do grau de dependência de pacientes após acidente vascular encefálico (AVE) na carga de trabalho de enfermagem requerida durante a internação em Unidade de Terapia Intensiva (UTI).

**Método:**

Coorte retrospectiva de pacientes pós-AVE admitidos entre 2019–2022 em uma UTI privada da cidade de São Paulo, Brasil. A gravidade neurológica foi mensurada pela *National Institutes of Health Stroke Scale (NIHSS)*, o nível de dependência pelo Índice de Katz e a carga de trabalho de enfermagem pelo *Nursing Activities Score* (NAS). As informações dos 98 pacientes foram coletadas em prontuários eletrônicos e o modelo de efeitos mistos foi aplicado nas análises.

**Resultados:**

Em uma amostra de pessoas idosas (76,1 ± 14,8 anos) e predominantemente masculina (62,4%), observou-se que a gravidade neurológica (p < 0,001) e o grau de dependência (p = 0,002) predizem, de forma independente, os valores do NAS, sem relação com o momento em que esta variável foi mensurada (admissão, dia médio de internação ou alta da UTI).

**Conclusão:**

A gravidade neurológica e o grau de dependência influenciam a carga de trabalho de enfermagem requerida por pacientes pós-AVE na UTI e devem ser considerados no dimensionamento da equipe.

## INTRODUÇÃO

Anualmente, os eventos cerebrovasculares são responsáveis por aproximadamente sete milhões de óbitos no mundo, sendo a segunda causa de morte nos países em desenvolvimento^(1)^. No Brasil, o acidente vascular encefálico (AVE), um dos mais frequentes eventos cerebrovasculares, é a principal causa de incapacitação na população com mais de 50 anos e representa 32,6% dos óbitos por doenças vasculares^(2)^.

O indivíduo acometido por AVE sofre uma série de alterações em seu metabolismo, sendo muito importante, ainda na fase aguda da doença, mensurar a gravidade do evento, prever e evitar possíveis sequelas^(3)^. Para isso, existem escalas preditoras de gravidade neurológica, com destaque para a *National Institutes of Health Stroke Scale* (NIHSS) que utiliza como parâmetros para o cálculo a idade do paciente e o seu comprometimento neurológico^(4)^.

O escore da NIHSS pode variar de 0 a 42 pontos, sendo que pontuações inferiores a 8 estão associadas a déficits neurológicos leves e transitórios, entre 8 e 17 correspondem a um evento moderado e escores superiores ou igual a 18 correspondem a déficit neurológico grave^(4)^. Uma das principais consequências desses eventos cerebrovasculares é o potencial de gerar importantes graus de incapacidade, já que aproximadamente 70% das pessoas acometidas ficam incapacitadas de retornar às suas atividades laborais e 50% têm dificuldades em realizar atividades de vida diária (AVDs)^(5)^.

Nessa perspectiva, um dos instrumentos utilizados na prática clínica para a avaliação das atividades básicas de vida diária (AbVDs) é o Índice de Katz, que permite identificar o grau de dependência ou incapacidade do paciente por meio da avaliação de seis funções básicas de autocuidado: banhar-se, vestir-se, alimentar-se, usar o banheiro, realizar transferências e manter controle sobre as eliminações, classificando o indivíduo como independente, dependente parcial ou dependente total para realizar tais ações^(6,7)^.

Pacientes com afecções neurológicas graves, como o AVE, são considerados neurocríticos e comumente necessitam de cuidados especializados oferecidos em Unidades de Terapia Intensiva (UTI). Neste contexto, o grau de dependência e a gravidade destes pacientes durante o tratamento intensivo pode intensificar a necessidade de cuidados prestados pela equipe de Enfermagem. Assim, torna-se evidente a importância da atuação da equipe de enfermagem na linha de cuidado ao doente com AVE, uma vez que os serviços prestados por esses profissionais são essenciais em todo o processo patológico. A atuação dessa categoria contribui para a minimização dos danos causados pelas alterações nas funções sensitivo-motoras durante o cuidado na fase aguda e na recuperação da doença, promovendo melhor independência funcional, incluindo a capacidade de realizar AbVDs e, consequentemente, proporcionando melhor qualidade de vida^(8,9)^.

Desse modo, muitos indivíduos que sofrem déficits neurológicos graves após o AVE apresentam incapacidade para realizar AbVDs, assim como gerenciar o autocuidado^9^, e estudos evidenciam a importância das intervenções de enfermagem durante todas as etapas de tratamento desses pacientes para o alcance de desfechos satisfatórios^(8,10,11)^. Pesquisas retratam o impacto do AVE na necessidade de cuidados de enfermagem requeridos pelos pacientes após a alta hospitalar^(11,12)^. Entretanto, poucos estudos investigaram o uso do NAS em pacientes neurológicos e os resultados encontrados indicam relação direta entre a gravidade da doença e a carga de trabalho de enfermagem^(10,13)^. Pacientes neurológicos com maior grau de criticidade e aqueles que podem evoluir para óbito requerem mais cuidado à beira-leito e terapias complexas, aumentando a carga de trabalho da equipe de enfermagem^(10)^.

Portanto, a literatura evidencia, de forma ampla, a aplicabilidade do NAS em pacientes neurológicos^(10,13)^, independentemente da condição que os levou à UTI, como no caso do AVE. Assim, os resultados do presente estudo podem ser de extrema relevância para o planejamento da assistência direcionada às necessidades especiais desse grupo durante o tratamento intensivo, além de proporcionar informações essenciais ao dimensionamento adequado da equipe de enfermagem para um cuidado seguro e eficaz.

Diante disso, questiona-se: a gravidade neurológica (NHISS) do paciente com AVE influencia a carga de trabalho de enfermagem durante a internação na UTI? O grau de dependência do paciente para realização das AbVDs impacta na requisição de cuidados assistenciais?

Nesse sentido, o objetivo do estudo foi verificar a influência da gravidade neurológica e do grau de dependência de pacientes pós AVE na carga de trabalho de enfermagem requerida durante a internação na UTI.

## MÉTODO

### Tipo do Estudo

Trata-se de uma coorte retrospectiva que analisou pacientes com AVE atendidos na emergência de um hospital privado da cidade de São Paulo, capital, no período de janeiro de 2019 a dezembro de 2022. O local do estudo consiste em um complexo hospitalar de caráter privado localizado na cidade de São Paulo, Brasil, composto por aproximadamente 50 leitos ativos de UTI geral, distribuídos entre 3 alas, sendo uma delas destinada a pacientes com acometimento neurológico.

### População

A população do estudo foi composta por 198 pacientes admitidos, no período do estudo, via pronto atendimento, e que tiveram, por meio de exame de neuroimagem, quadro confirmado de AVE isquêmico ou hemorrágico na emergência da instituição e foram encaminhados para a UTI neurológica.

### Definição da Amostra

A partir da lista fornecida pelo departamento de protocolos institucionais referente aos 198 pacientes admitidos no pronto-atendimento, foi realizado um cálculo amostral, considerando um modelo com tamanho de efeito η^2^ parcial = 0.05. Para isso utilizou-se o modelo ANOVA de medidas repetidas, o mais próximo ao modelo de efeitos mistos para dimensionar amostras. Com erros tipo I e II de 5% cada, conclui-se que seria necessário observar ao menos 72 sujeitos. No entanto, para compensar possíveis perdas, foi prevista uma adição de 30%, o que resultou em uma amostra final estimada em 93 participantes. A análise pós-hoc para avaliar o poder alcançado indicou um poder de estudo (probabilidade 1 erro β) de 0,80, calculado para um tamanho de efeito de 0,30 em uma amostra de 93 participantes.

Foram incluídos no estudo os indivíduos admitidos no pronto-atendimento em questão, no período de janeiro de 2019 a dezembro de 2022, com quadro de AVE confirmado por neuroimagem, que possuíam idade maior ou igual a 18 anos e que foram internados na UTI neurológica após manejo inicial. As exclusões de indivíduos da amostra ocorreram mediante ausência do NIHSS calculado na admissão da emergência, permanência na UTI por um período menor que 24 horas e falta de informações registradas no prontuário eletrônico quanto às variáveis de interesse do estudo.

### Variáveis do Estudo

As variáveis independentes do estudo foram a gravidade neurológica segundo o NIHSS^4^ atribuído e o grau de dependência de pacientes para as AbVDs, de acordo com o Índice de Katz^7^. A NIHSS utiliza como parâmetros a idade do indivíduo, nível de consciência, linguagem, perda de campo visual, movimentos extraoculares, força motora, ataxia, disartria e perda sensorial. Seu escore varia entre 0 e 42 pontos, na qual as maiores pontuações estão relacionadas com menores chances de resultados positivos no pós-evento^(6,7)^.

O Índice de Katz foi utilizado em sua forma categórica, atribuindo para cada item uma pontuação da seguinte forma: 0- para os participantes que possuem total dependência (dependente); 0,5 – pacientes que necessitam de auxílio parcial (parcialmente dependentes) e 1- pacientes que realizam atividade sem auxílio (independentes). A pontuação final resultante ficou entre 0–6 pontos^(7)^.

Já a carga de trabalho de enfermagem, variável dependente, foi identificada pelo *Nursing Activities Score* (NAS), cujo escore final (máximo de 176,8%) reflete a porcentagem de tempo, durante 24 horas, que o paciente necessitou da assistência do profissional de enfermagem, por meio da análise de 23 intervenções de enfermagem^(14)^.

Como variáveis secundárias, foram avaliadas características dos pacientes (sexo, idade, cor da pele, situação conjugal, religião, nível de escolaridade, índice de massa corporal – IMC, índice de comorbidade de Charlson), tipo de AVE (isquêmico ou hemorrágico), suporte utilizado na terapia intensiva, indicadores de gravidade fisiológica, como *Sequential Organ Failure Assessment* (SOFA) e *Simplified Acute Physiology Score III* (SAPS 3) na admissão na UTI, escala de Rankin pré-AVC, além de dados clínicos como tempo de internação na UTI e no hospital e desfecho hospitalar (sobrevivente ou não sobrevivente).

### Coleta de Dados

O presente estudo foi realizado por meio de coleta de dados dos prontuários eletrônicos dos pacientes e do sistema Epimed Solutions® entre os meses de agosto e setembro de 2023. Informações adicionais foram fornecidas pelo grupo de protocolos institucionais (Desfecho Clínico), setor que acompanha o acionamento e execução de protocolos assistenciais, como no AVE, e registra o desfecho de cada paciente.

Por meio da aplicação de um instrumento de coleta de dados elaborado pelos pesquisadores, foram extraídos os acionamentos de AVE realizados via pronto-atendimento e desses, analisadas as informações dos pacientes que tiveram diagnóstico confirmado com encaminhamento à UTI, além da pontuação do admissional do NIHSS realizada pelo neurologista de plantão, conforme protocolo institucional. Ademais, foram coletados os itens pontuados do NIHSS para identificar os déficits presentes em cada paciente no momento da admissão no pronto-atendimento.

Para registro do nível de dependência para AbVD, foi utilizado o Índice de Katz em três momentos: estado prévio à UTI por meio de informações fornecidas por familiares/cuidadores, admissão na UTI e na saída da unidade. O preenchimento do índice de Katz (admissão e saída da UTI) foi baseado nos registros da equipe de enfermagem, os quais estavam relacionados aos cuidados diários realizados no grupo em questão. Além disso, com o objetivo de completar essa informação, buscou-se associar com as avaliações da equipe de fisioterapia que aplica o índice de Katz diariamente durante a avaliação funcional dos pacientes e é treinada para a aplicabilidade do índice durante o período de contratação institucional.

O NAS, coletado do prontuário eletrônico, foi calculado pelos enfermeiros da UTI em questão, diariamente, às 17 horas. Assim, foram registrados para este estudo o NAS em três momentos de permanência do paciente na UTI: admissão, intermediário (dia médio de internação) e na saída da unidade.

Por fim, com o objetivo de garantir a confiabilidade dos dados extraídos e reduzir vieses de seleção e de reporte de dados, estes foram coletados por um único pesquisador, utilizando um instrumento de coleta padronizado. Além disso, os prontuários que não continham todas as variáveis reportadas tiveram seus números de atendimento, utilizados para sua localização, excluídos do banco de dados e, consequentemente, do estudo.

### Análise e Tratamento dos Dados

Os dados foram analisados por meio do pacote estatístico R versão 4.3.0. As variáveis contínuas foram apresentadas por média, desvio padrão, mediana e quartis e, as categóricas, como frequência absoluta (n) e relativa (%). Para testar a influência das variáveis independentes (gravidade neurológica e nível de dependência) na carga de trabalho de enfermagem, foi utilizado o modelo de efeitos mistos, empregando o teste Qui-quadrado de Wald tipo III. Esta metodologia permitiu avaliar as medições de um paciente ao longo do período estudado, bem como sua variação. O nível de significância adotado foi de 0,05, em testes bicaudais, considerado um α de 5%.

### Aspectos Éticos

O presente estudo seguiu os princípios éticos que regulamentam as pesquisas envolvendo seres humanos de acordo com as Resoluções nº 466/12 e 510/16 do Conselho Nacional de Saúde. Conforme preconizado pela instituição, inicialmente o projeto foi autorizado pelas coordenações das unidades onde a pesquisa seria conduzida e submetido à Central de Apoio ao Pesquisador (AVAP), plataforma de gerenciamento de pesquisas do Hospital onde o estudo foi realizado. Após, o projeto foi submetido ao Comitê de Ética em Pesquisa (CEP), com posterior aprovação (Parecer nº 6.176.270 e CAAE nº 70539323.0.0000.5461). O CEP autorizou a dispensa da aplicação de Termo de Consentimento Livre e Esclarecido aos pacientes, visto que a coleta de dados foi realizada de forma secundária por meio do sistema Epimed Solutions® e dos prontuários eletrônicos.

## RESULTADOS

Ao analisarmos o perfil dos pacientes incluídos no presente estudo, identificou-se o predomínio do sexo masculino (n = 58; 62,4%), cor da pele branca (n = 88; 94,6%), estado civil de casado (n = 60; 64,5%), ensino superior completo (n = 70; 75,3%) e religião católica (n = 58; 62,3%). A idade média foi de 76,1 ± 14,8 anos, mediana de 81 anos e o índice de Massa Corporal (IMC) médio de 27,4 ± 4,7.

Houve predomínio de pacientes com AVE de origem isquêmica (95,7%), com admissão na UTI por causa clínica (94,6%), procedentes da emergência (82,8%), independentes a partir da avaliação clínica (79,6%) e com ausência de sintomas incapacitantes (46,2%) segundo a escala Rankin pré-AVC.

Quanto ao suporte utilizado na UTI, 12,9% da amostra necessitou de ventilação mecânica e 4,3% evoluiu para traqueostomia. O uso de vasopressores foi registrado em 17,2% da amostra, apenas 2,1% necessitaram de terapia de substituição renal e não houve a utilização de oxigenação por membrana extracorpórea.

A média e a mediana da pontuação do NHISS na admissão do pronto-atendimento foi de 8,5 ± 6,2 e 7, respectivamente, e identificou-se maior frequência de déficits relacionados à paralisia facial central (29%), sensibilidade (26,9% perda leve a moderada e 12,9% com perda grave) e capacidade da fala, evidenciada pela disartria (25,8% apresentando disartria leve a moderada e 23,7% com disartria grave) e melhor linguagem (16,1% com afasia grave). Os demais achados relacionados ao índice de comorbidade, gravidade e tempo de internação na UTI e no hospital estão representados na Tabela 1.

**Tabela 1:** Caracterização da amostra segundo índice de comorbidade de Charlson, gravidade e tempo de internação – São Paulo, SP, Brasil 2023.

	Média	DP	Mín	Q1	Mediana	Q3	Máx	IC min.	IC max.
NIHSS	8,5	6,2	1,0	3,0	7,0	14	24	7,3	9,8
Charlson	1,4	1,6	0,0	0,0	1,0	2,0	6,0	1,2	1,8
SAPS 3	51,3	8,3	34,0	46,0	54,0	56,0	82,0	49,6	53,0
SOFA	2,2	2,6	0,0	0,0	2,0	3,0	13,0	1,8	2,8
Tempo de internação em UTI	5,6	6,0	1,0	2,0	3,0	7,0	40,0	4,6	7,2
Tempo de internação	19,5	19,1	2,0	8,0	12,0	23,0	107,0	16,2	24,1
Hospitalar									
Índice de Katz									
Prévio	5,3	1,1	1,5	5,0	6,0	6,0	6,0	5,0	5,5
Admissão	3,1	1,8	0,5	1,0	3,0	4,5	6,0	2,7	3,4
Alta	3,2	2,1	0,0	2,0	3,0	4,5	6,0	2,8	3,6
NAS									
Admissão	50,8	20,3	24,8	42,7	49,4	71,3	108,7	54,1	62,3
Intermediário	56,5	17,3	31	42,3	51,6	67,8	101,1	53,2	60,2
Alta	51,7	14,7	26,6	40,8	49,3	61,5	88,5	48,9	54,8

Mín – Mínimo; Máx – Máximo; Q1 – Quartil 1; Q3 – Quartil 3; IC – Intervalo de Confiança; NIHSS – *National Institute of Health Stroke Scale*; Charlson – *Charlson Comorbidity Index*; SAPS3 – *Simplified Acute Physiology Score 3;* SOFA – *Sequential Sepsis-related Organ Failure Assessment*; UTI – Unidade de Terapia Intensiva; NAS – *Nursing Activities Score*; Katz – *Índice de Katz*.

Quanto ao Índice de Katz coletado com familiares, os pacientes apresentavam valor médio do estado prévio de 5,3, refletindo independência no grupo. Após o evento cerebrovascular, o valor médio reduziu de forma significativa para 3,1, com discreto aumento no momento da alta da UTI para 3,2.

No que se refere ao NAS, observou-se redução entre os momentos de mensuração no decorrer da internação na UTI. No momento da admissão na unidade crítica, foram observados valores mais altos do NAS, com média de 58,0% e mediana de 49,4%, na coleta intermediária notou-se discreta mudança nos escores, 56,5% e 51,6%, enquanto na alta, a redução do NAS se manteve, 51,7% e 49,3%, respectivamente.

Quanto à categorização da dependência a partir do Índice de Katz, foi observado que o nível de dependência se eleva em todos os itens analisados no momento da admissão na UTI em comparação ao comportamento do paciente antes do evento cerebrovascular (estado prévio). Entretanto, observa-se melhora dos percentuais no momento da alta, com exceção do item “transferência”, que apresenta aumento exponencial da dependência de 1,1% na admissão para 30,1% na alta da UTI.

Em relação aos itens do NAS, observou-se frequência significativa de intervenções relacionadas aos cuidados básicos, como no item 4a (realização de procedimentos de higiene), com 71% na admissão na UTI, seguido de 68,8% no intermediário e, por fim, 76,3% na alta. O mesmo aplicou-se ao item 6b, necessidade de mobilização e posicionamento mais do que 3 vezes em 24 horas ou com dois profissionais de enfermagem, pontuando 57,7%, 62,4% e 39,8%, de forma respectiva aos momentos citados. Outras intervenções com achados mais expressivos estavam relacionadas à monitorização e controles (item 1a); investigações laboratoriais, bioquímica e microbiológica (item 2); administração de medicamentos, exceto drogas vasoativas (item 3), suporte e cuidado aos familiares (item 7a), tarefas administrativas e gerenciais de rotina (item 8a) e medida quantitativa de débito urinário (item 17). A Figura 1 mostra as pontuações dos itens do NAS nos três momentos mensurados na UTI.

**Figura 1:** Frequência de itens do NAS pontuados nos diferentes momentos de mensuração.

A Tabela 2 evidencia a relação entre a pontuação do NHISS no momento da admissão do pronto atendimento e a demanda de cuidados de enfermagem mensurada pelo NAS nos três momentos (admissão, intermediário e alta). Nota-se que cada unidade do NIHSS aumenta, aproximadamente, 1,5 pontos no NAS na admissão, 1,3 no dia médio de internação (intermediário) e 0,7 na alta da UTI (p = 0,013).

**Tabela 2:** Pontuação do NHISS e valores do NAS nos diferentes momentos de mensuração na UTI – São Paulo, SP, Brasil, 2023.

	Momento (NAS)	Coeficiente	DP	IC mín.	IC máx.	p-Valor
	Admissão	1,589	0,267	1,061	2,116	0,013
NHISS	Intermediário	1,335	0,267	0,808	1,863	
	Alta	0,767	0,267	0,240	1,294	

DP – Desvio padrão; IC mín – Intervalo de confiança mínimo; IC Máx – Intervalo de confiança máximo.

A Figura 2 evidencia que quanto maior o valor do NIHSS, maior a média marginal estimada no NAS, independente no momento analisado. Ademais, observa-se o quanto a demanda de cuidados requerida pelo paciente muda em relação a sua pontuação NHISS admissional e que os pacientes com NHISS mais elevados evoluíram com queda significativamente (p = 0,013) mais acentuada do valor do NAS no decorrer da internação, diferente daqueles admitidos na UTI com valores no NHISS mais baixos, que apresentam menor variação das pontuações do NAS registradas. Comportamento semelhante também foi identificado entre o Índice de Katz e o NAS dos pacientes analisado nos três momentos de internação na UTI (p = 0,328).

**Figura 2:** Gráficos de relação entre o escore NAS frente ao NIHSS e Índice de Katz nos diferentes momentos de mensuração na UTI.

A Tabela 3 apresenta a análise da influência do NHISS e do Índice de Katz na carga de trabalho de enfermagem por meio do NAS mensurado nos três momentos. Nota-se que o NIHSS (p < 0,001) e o Índice de Katz (p = 0,002) predizem os valores do NAS de forma independente e não há relação com o momento em que a carga de trabalho de enfermagem é mensurada. Assim, pacientes mais dependentes para AbVDs (valores do Índice de Katz mais baixos) e aqueles com maior gravidade neurológica observada pelo NIHSS terão escore NAS mais elevados, independente do momento de mensuração (admissão, intermediário ou alta).

**Tabela 3:** Influência do Índice de Katz e do NHISS na carga de trabalho mensurada pelo NAS na UTI nos diferentes momentos – São Paulo, SP, Brasil, 2023.

	X^2^	p-Valor (>X^2^)
(Intercepto)	218,862	<0,001
Momento da mensuração	0,385	0,535
NIHSS	13,615	<0,001
Katz	10,002	0,002
Momento:NIHSS	3,366	0,067
Momento:Katz	0,957	0,328

X^2^ – qui-quadrado de Pearson; NIHSS – *National Institute of Health Stroke Scale*; Katz – *Índice de Katz.*

Por fim, a maioria dos pacientes teve alta da UTI (94,6%) e sobreviveu (91,4%) durante a internação hospitalar.

## DISCUSSÃO

O presente estudo evidenciou que o NIHSS e o índice de Katz influenciam os valores do NAS de forma independente, sem relação com o momento de mensuração da carga de trabalho de enfermagem na UTI (admissão, intermediário ou alta).

A amostra foi composta predominantemente por homens e idosos. Nesse sentido, sabe-se que doenças crônicas associadas a fatores como idade avançada e internação em UTI afetam negativamente a independência funcional dos indivíduos, de modo que, na medida em que ocorre o aumento da incidência de doenças, maior é o risco de incapacitação nas atividades básicas de vida^(15)^. Dessa forma, é válido ressaltar que os eventos cerebrovasculares possuem associação direta com o grau de dependência de idosos^(16)^. Assim, justifica-se o fato de os pacientes apresentarem, previamente ao AVE, maior grau de independência funcional com posterior decréscimo do escore no índice de Katz, tornando-se mais dependentes após o evento cerebrovascular.

Junto a isso, o conjunto de déficits neurológicos apresentados pelos pacientes em consequência de alterações vasculares está intimamente relacionado com a sua gravidade. Resultados presentes na literatura demonstraram a existência de relação entre sintomas leves de AVE, apontados pela escala NHISS, e a expansão ou área de lesão neurológica^(16)^. Considerando que as pontuações entre 8 e 17 pontos caracterizam gravidade neurológica moderada, o presente estudo evidenciou pontuação média de 8,5 do NHISS associada à frequência maior de déficits relacionados à paralisia facial central, sensibilidade e capacidade da fala, conforme apontado pelos índices de disartria e melhor linguagem, além de alterações em força motora.

Alterações referentes à linguagem, negligência, déficits visuais, atividades motoras e sensoriais, geralmente presentes em casos considerados graves, estão frequentemente relacionadas a desfechos menos favoráveis e, consequentemente, maior grau de dependência após o evento neurológico^(17)^. Dessa forma, a presença de tais déficits presentes na amostra em questão pode estar relacionada a mudanças nos graus de dependência para execução das AbVDs.

Ademais, sabe-se que o principal motivo das internações de idosos na UTI é a agudização de doenças crônicas, principalmente de origem vascular, como ocorre no AVE^(17,18)^. A assistência de enfermagem prestada a esta população está associada ao aumento da carga de trabalho de enfermagem^(19)^, conforme evidenciado pelas pontuações médias do NAS na admissão na UTI (58,0%), no momento intermediário (56,5%) e na alta (51,7%). Achados semelhantes foram encontrados em estudo desenvolvido por Lucchini et al.^(19)^, que registrou NAS médio de 59,33 em UTIs da Itália, assim como em estudo brasileiro que analisou idosos que necessitaram de cuidados intensivos^(20)^.

Diante disso, em consequência de alterações fisiológicas causadas pelo próprio processo de envelhecimento, muitas vezes associadas a determinados graus de fragilidade, esse público torna-se mais vulnerável, fato esse frequentemente potencializado pelas condições patológicas que os cercam, como, por exemplo, incapacitações causadas pelo AVE^(21,22)^. No tocante ao grupo em questão, foi perceptível um predomínio de cuidados referentes às atividades básicas, como mobilização/posicionamento e procedimentos de higiene, incluindo banho no leito, presentes durante toda a permanência na UTI. Geralmente, atividades que envolvem higiene e mobilização estão associadas a perda progressiva de autonomia para realização de atividades básicas e instrumentais, essas acentuadas pelos variados graus de déficits provocados pelo AVE^(8)^.

Outras intervenções com maior frequência em todos os momentos da internação relacionavam-se com a necessidade de monitorização e sinais vitais (item 1), investigação microbiológica (item 2), medicação, exceto drogas vasoativas (item 3), e suporte e cuidado aos familiares (item 7). Esses achados justificam-se mais uma vez pela faixa etária predominante no grupo, que necessita de maior monitorização contínua, em consequência das modificações sofridas pelo organismo, essas afetadas, principalmente, pelas condições de saúde que impactam diretamente o uso de medicações de uso contínuo, visando controle de tais condições, e geralmente resultando na chamada polifarmácia^(23,24)^.

Diante das propriedades psicométricas do Índice de Katz e suas versões, identificou-se que ele é um instrumento válido e confiável para se determinar e avaliar a capacidade para realizar AbVDs e mortalidade de pacientes pós-AVE^(8,25)^. Nesse sentido, o estudo em questão demonstrou relação da gravidade neurológica com a capacidade de realização das AbVDs, quando comparadas ao grau funcional prévio. Com uma interação representada por coeficientes negativos, observou- se que após o evento cerebrovascular houve uma influência do AVE sob a capacidade funcional na seguinte proporção: reduziu-se 0,299 do Katz admissional e 0,332 do desfecho em relação a sua capacidade prévia, aumentando o nível de dependência da amostra.

Somado a isso, evidências apontam a influência direta do AVE como agente incapacitante para funcionalidade e independência física de um indivíduo, demonstrando relação direta e aceitável com o índice de Katz, considerado excelente preditor para incapacitações pós-evento, especialmente ao associar idades mais elevadas com maiores graus de incapacidade^(26)^. Nesse sentido, o estudo em pauta mostra interação direta entre a ocorrência do AVE e a alteração em sua capacidade funcional. Sendo frequente a existência de interações entre a gravidade da doença associada a carga de trabalho de enfermagem, sabe-se que a escala NHISS atua como um indicador de gravidade neurológica, geralmente relacionado à extensão e gravidade do evento cerebrovascular. O presente estudo evidenciou que pacientes que apresentavam maior gravidade possuíam inicialmente escores de maior demanda assistencial, assim como uma queda mais acentuada desse índice durante o processo de internação na UTI. Achados presentes na literatura demonstraram a existência dessa relação de forma semelhante, ao evidenciar em uma UTI neurológica a presença de maiores pontuações NAS associadas a pacientes que apresentam maior gravidade pelo SAPSII^(13)^.

Desse modo, sabe-se que a gravidade da doença exerce influência sobre os cuidados prestados ao paciente e que, junto a isso, a sua dependência física irá afetar a realização de determinadas atividades. No entanto, a gravidade da doença não deve ser o único fator a ser considerado para determinar os cuidados de enfermagem prestados, uma vez que, diante da pluralidade apresentada pelos pacientes, outros fatores, como dependência funcional e nível de consciência, devem ser considerados^(8,19,27)^. Nesse sentido, foi então evidenciada interação, de forma independente, entre as variáveis citadas (NHISS e Índice de Katz) com o NAS, inferindo-se que tanto a gravidade neurológica quanto o grau de incapacitação irão influenciar a demanda de cuidados de enfermagem requerida pelos pacientes na UTI, independente do momento de mensuração. Contudo, o estudo apresenta como limitações: a presença de uma amostra pertencente a uma única instituição de saúde, com uma população com acesso a saúde suplementar e maior acompanhamento clínico de longo prazo, o que pode limitar sua validade externa. Além disso, destaca-se a ausência de uma análise segregada do grupo estudado por tipo de evento (isquêmico ou hemorrágico), sendo os dados apresentados de forma unificada.

Por outro lado, destaca-se a inovação desta pesquisa, uma vez que não existem trabalhos nesta linha, ou seja, que apontem a relação entre o distúrbio neurológico abordado e a demanda de cuidados de enfermagem em terapia intensiva. Junto a isso, destaca-se a boa estrutura do delineamento estabelecido para análise dos dados, o que traz propriedade aos achados presentes neste estudo.

Por fim, considerando que a carga de trabalho de enfermagem interfere diretamente na qualidade do cuidado^(28)^, os resultados aqui apresentados possuem grande potencial para agregar à prática clínica e pesquisa, uma vez que destacam, com ineditismo, a assistência da enfermagem intensiva prestada aos pacientes com diagnóstico clínico AVE. Desse modo, as evidências referentes à carga de trabalho requerida por pacientes pós-AVE internados na UTI contribuem para o fortalecimento da gestão em saúde ao identificar aspectos que podem auxiliar no dimensionamento da equipe de enfermagem e distribuição diária de paciente(s) por profissional pautados na gravidade neurológica, no grau de dependência e outras condições pertinentes para garantia da segurança da assistência intensiva prestada.

Embora esse estudo tenha sido conduzido com um número amostral superior ao estimado pelo cálculo estatístico, acredita-se que a realização de novas investigações sobre o NAS em pacientes com AVE, especialmente multicêntricas e em outros contextos de saúde, como instituições públicas e unidades não críticas, podem fortalecer e fundamentar a prática assistencial a essa população por meio de consolidação de mais evidências científicas.

## CONCLUSÃO

Diante do exposto, conclui-se que o grau de gravidade neurológica apresentado pela pontuação NHISS e de dependência funcional segundo Índice de Katz apresentados pelos pacientes pós-AVE influenciam significativamente a carga de trabalho de enfermagem mensurada pelo NAS, independente do momento de sua mensuração durante a internação na UTI.

## Data Availability

Todo o conjunto de dados que dá suporte aos resultados deste estudo está disponível mediante solicitação ao autor correspondente, Felipe da Cruz Lima. O conjunto de dados não está publicamente disponível devido os resultados que compõem a pesquisa se tratam de informações que comprometem a privacidade dos participantes da pesquisa.

# Translation: Influencia de la gravedad neurológica y el grado de dependencia de los pacientes tras un accidente cerebrovascular en la carga de trabajo de enfermería: cohorte retrospectiva

**DOI:** 10.1590/1980-220X-REEUSP-2024-0302es

**Authors:** Felipe da Cruz Lima, Joathan Borges Ribeiro, Samia Denadai, Lilia Souza Nogueira, Renata Eloah de Lucena Ferretti-Rebustini

**Affiliations:** 1 Universidade de São Paulo, Escola de Enfermagem, São Paulo, SP, Brasil.; 2 Hospital Sírio-Libanês, Unidade de Terapia Intensiva, São Paulo, SP, Brasil.; 3 Universidade de São Paulo, Escola de Enfermagem, Enfermagem Médico-Cirúrgica, São Paulo, SP, Brasil.

**Keywords:** Accidente Cerebrovascular, Enfermería, Estado Funcional, Carga de Trabajo

## Abstract

**Objetivo:**

Verificar la influencia de la gravedad neurológica y el grado de dependencia de los pacientes tras un accidente cerebrovascular (ACV) en la carga de trabajo de enfermería requerida durante la hospitalización en una Unidad de Terapia Intensiva (UTI).

**Método:**

Cohorte retrospectiva de pacientes post-ACV ingresados entre 2019 y 2022 en una UTI privada de la ciudad de São Paulo, Brasil. La gravedad neurológica se midió mediante la *Escala del Ictus del Instituto Nacional de Salud (NIHSS)*, el nivel de dependencia mediante el Índice de Katz y la carga de trabajo de enfermería mediante la *Puntuación de Actividades de Enfermería* (NAS). La información de los 98 pacientes se recopiló en historias clínicas electrónicas y se aplicó el modelo de efectos mixtos en los análisis.

**Resultados:**

En una muestra de personas mayores (76,1 ± 14,8 años) y predominantemente masculinas (62,4 %), se observó que la gravedad neurológica (p < 0,001) y el grado de dependencia (p = 0,002) predicen, de forma independiente, los valores del NAS, sin relación con el momento en que se midió esta variable (ingreso, día medio de hospitalización o alta de la UTI).

**Conclusión:**

La gravedad neurológica y el grado de dependencia influyen en la carga de trabajo de enfermería requerida por los pacientes post-ACV en la UTI y deben tenerse en cuenta a la hora de dimensionar el equipo.

## INTRODUCCIÓN

Cada año, los accidentes cerebrovasculares son responsables de aproximadamente siete millones de muertes en todo el mundo, siendo la segunda causa de muerte en los países en desarrollo^(1)^. En Brasil, el accidente cerebrovascular (ACV), uno de los accidentes cerebrovasculares más frecuentes, es la principal causa de incapacitación en la población mayor de 50 años y representa el 32,6 % de las muertes por enfermedades vasculares^(2)^.

Las personas afectadas por un ACV sufren una serie de alteraciones en su metabolismo, por lo que es muy importante, incluso en la fase aguda de la enfermedad, medir la gravedad del evento, prever y evitar posibles secuelas^(3)^. Para ello, existen escalas predictivas de gravedad neurológica, entre las que destaca la *National Institutes of Health Stroke Scale* (NIHSS por su sigla en inglés), que utiliza como parámetros para el cálculo la edad del paciente y su compromiso neurológico^(4)^.

La puntuación de la NIHSS puede variar de 0 a 42 puntos, y las puntuaciones inferiores a 8 se asocian con déficits neurológicos leves y transitorios, entre 8 y 17 corresponden a un evento moderado y las puntuaciones superiores o iguales a 18 corresponden a un déficit neurológico grave^(4)^. Una de las principales consecuencias de estos eventos cerebrovasculares es el potencial de generar grados importantes de discapacidad, ya que aproximadamente el 70 % de las personas afectadas quedan incapacitadas para volver a sus actividades laborales y el 50 % tienen dificultades para realizar las actividades de la vida diaria (AVD)^(5)^.

En esta perspectiva, uno de los instrumentos utilizados en la práctica clínica para evaluar las actividades básicas de la vida diaria (ABVD) es el Índice de Katz, que permite identificar el grado de dependencia o incapacidad del paciente mediante la evaluación de seis funciones básicas de autocuidado: bañarse, vestirse, alimentarse, ir al baño, realizar transferencias y controlar las eliminaciones, clasificando al individuo como independiente, parcialmente dependiente o totalmente dependiente para realizar dichas acciones^(6,7)^.

Los pacientes con afecciones neurológicas graves, como el ictus, se consideran neurocríticos y suelen necesitar cuidados especializados que se ofrecen en las unidades de terapia intensiva (UTI). En este contexto, el grado de dependencia y la gravedad de estos pacientes durante el tratamiento intensivo pueden intensificar la necesidad de cuidados prestados por el equipo de enfermería. Por lo tanto, se hace evidente la importancia del papel del equipo de enfermería en la atención al paciente con ictus, ya que los servicios prestados por estos profesionales son esenciales en todo el proceso patológico. La actuación de esta categoría contribuye a minimizar los daños causados por las alteraciones en las funciones sensitivo-motoras durante los cuidados en la fase aguda y en la recuperación de la enfermedad, promoviendo una mayor independencia funcional, incluida la capacidad de realizar AVD, y, en consecuencia, proporcionando una mejor calidad de vida^(8,9)^.

De este modo, muchas personas que sufren déficits neurológicos graves tras un ictus presentan incapacidad para realizar AVD, así como para gestionar el autocuidado^(9)^, y los estudios evidencian la importancia de las intervenciones de enfermería durante todas las etapas del tratamiento de estos pacientes para alcanzar resultados satisfactorios^(8,10,11)^. Las investigaciones reflejan el impacto del ictus en la necesidad de cuidados de enfermería que requieren los pacientes tras el alta hospitalaria^(11,12)^. Sin embargo, pocos estudios han investigado el uso de la NAS en pacientes neurológicos y los resultados encontrados indican una relación directa entre la gravedad de la enfermedad y la carga de trabajo de enfermería^(10,13)^. Los pacientes neurológicos con mayor grado de gravedad y aquellos que pueden evolucionar hacia la muerte requieren más cuidados a pie de cama y terapias complejas, lo que aumenta la carga de trabajo del equipo de enfermería^(10)^.

Por lo tanto, la literatura evidencia ampliamente la aplicabilidad de la NAS en pacientes neurológicos^(10,13)^, independientemente de la condición que los llevó a la UTI, como en el caso del ACV. Así, los resultados del presente estudio pueden ser de extrema relevancia para la planificación de la asistencia dirigida a las necesidades especiales de este grupo durante el tratamiento intensivo, además de proporcionar información esencial para la adecuada dotación del equipo de enfermería para una atención segura y eficaz.

Ante esto, se plantea la siguiente pregunta: ¿influye la gravedad neurológica (NHISS) del paciente con ACV en la carga de trabajo de enfermería durante la estadía en la UTI? ¿El grado de dependencia del paciente para realizar las AVD influye en la solicitud de cuidados asistenciales?

En este sentido, el objetivo del estudio fue verificar la influencia de la gravedad neurológica y el grado de dependencia de los pacientes post-ictus en la carga de trabajo de enfermería requerida durante la hospitalización en la UTI.

## MÉTODO

### Tipo de Estudio

Se trata de una cohorte retrospectiva que analizó a pacientes con ictus atendidos en el servicio de urgencias de un hospital privado de la ciudad de São Paulo, capital, en el periodo comprendido entre enero de 2019 y diciembre de 2022. El lugar del estudio es un complejo hospitalario privado situado en la ciudad de São Paulo, Brasil, que cuenta con aproximadamente 50 camas activas de UTI general, distribuidas en tres alas, una de ellas destinada a pacientes con afectación neurológica.

### Población

La población del estudio estuvo compuesta por 198 pacientes ingresados, durante el período del estudio, a través del servicio de urgencias, y que, mediante un examen de neuroimagen, presentaron un cuadro confirmado de ictus isquémico o hemorrágico en la sala de urgencias del centro y fueron derivados a la UTI neurológica.

### Definición de la Muestra

A partir de la lista proporcionada por el departamento de protocolos institucionales relativa a los 198 pacientes ingresados en urgencias, se realizó un cálculo muestral, considerando un modelo con un tamaño del efecto η^2^ parcial = 0,05. Para ello se utilizó el modelo ANOVA de medidas repetidas, el más cercano al modelo de efectos mixtos para dimensionar muestras. Con errores de tipo I y II del 5 % cada uno, se concluyó que sería necesario observar al menos 72 sujetos. Sin embargo, para compensar posibles pérdidas, se previó un aumento del 30 %, lo que dio como resultado una muestra final estimada de 93 participantes. El análisis post hoc para evaluar la potencia alcanzada indicó una potencia del estudio (probabilidad 1 error β) de 0,80, calculada para un tamaño del efecto de 0,30 en una muestra de 93 participantes.

Se incluyeron en el estudio los individuos admitidos en el servicio de urgencias en cuestión, en el periodo comprendido entre enero de 2019 y diciembre de 2022, con un cuadro de ictus confirmado por neuroimagen, que tenían una edad igual o superior a 18 años y que fueron ingresados en la UTI neurológica tras el tratamiento inicial. Se excluyó de la muestra a los individuos que no presentaban el NIHSS calculado al ingreso en urgencias, permanecieron en la UTI durante menos de 24 horas o no tenían información registrada en la historia clínica electrónica sobre las variables de interés del estudio.

### Variables del Estudio

Las variables independientes del estudio fueron la gravedad neurológica según la puntuación NIHSS^4^ asignada y el grado de dependencia de los pacientes para las AVD, según el índice de Katz^7^. El NIHSS utiliza como parámetros la edad del individuo, el nivel de conciencia, el lenguaje, la pérdida del campo visual, los movimientos extraoculares, la fuerza motora, la ataxia, la disartria y la pérdida sensorial. Su puntuación varía entre 0 y 42 puntos, en la que las puntuaciones más altas se relacionan con menores posibilidades de resultados positivos después del evento^(6,7)^.

El Índice de Katz se utilizó en su forma categórica, asignando a cada ítem una puntuación de la siguiente manera: 0 para los participantes que tienen dependencia total (dependientes); 0,5 para los pacientes que necesitan ayuda parcial (parcialmente dependientes) y 1 para los pacientes que realizan actividades sin ayuda (independientes). La puntuación final resultante fue de 0 a 6 puntos^(7)^.

Por su parte, la carga de trabajo de enfermería, variable dependiente, se identificó mediante el *Nursing Activities Score* (NAS), cuya puntuación final (máximo 176,8 %) refleja el porcentaje de tiempo, durante 24 horas, que el paciente necesitó la asistencia del profesional de enfermería, mediante el análisis de 23 intervenciones de enfermería^(14)^.

Como variables secundarias, se evaluaron las características de los pacientes (sexo, edad, color de piel, estado civil, religión, nivel de escolaridad, índice de masa corporal IMC, índice de comorbilidad de Charlson), tipo de ACV (isquémico o hemorrágico), soporte utilizado en terapia intensiva, indicadores de gravedad fisiológica, como la *Evaluación Secuencial de Fallo Orgánico* (*Sequential Organ Failure Assessmen t*-SOFA) y la *Puntuación Simplificada de Fisiología Aguda III* (*Simplified Acute Physiology Score III* SAPS 3) al ingreso en la UTI, la escala de Rankin previa al ACV, además de datos clínicos como el tiempo de estancia en la UTI y en el hospital y el resultado hospitalario (superviviente o no superviviente).

### Recolección de Datos

El presente estudio se realizó mediante la recolección de datos de los historiales electrónicos de los pacientes y del sistema Epimed Solutions® entre los meses de agosto y septiembre de 2023. Se obtuvo información adicional del grupo de protocolos institucionales (Resultado Clínico), sector que supervisa la activación y ejecución de los protocolos asistenciales, como en el caso del ictus, y registra el resultado de cada paciente.

Mediante la aplicación de un instrumento de recolección de datos elaborado por los investigadores, se extrajeron los casos de ACV atendidos en urgencias y, de estos, se analizó la información de los pacientes con diagnóstico confirmado y derivados a la UTI, además de la puntuación de admisión del NIHSS realizada por el neurólogo de guardia, según el protocolo institucional. Además, se recopilaron los ítems puntuados del NIHSS para identificar los déficits presentes en cada paciente en el momento del ingreso en urgencias.

Para registrar el nivel de dependencia para las ABVD, se utilizó el Índice de Katz en tres momentos: estado previo a la UTI mediante información proporcionada por familiares/cuidadores, ingreso en la UTI y al salir de la unidad. El llenado del índice de Katz (ingreso y alta de la UTI) se basó en los registros del equipo de enfermería, que estaban relacionados con los cuidados diarios realizados en el grupo en cuestión. Además, con el objetivo de completar esta información, se buscó asociarla con las evaluaciones del equipo de fisioterapia que aplica el índice de Katz diariamente durante la evaluación funcional de los pacientes y que está capacitado para la aplicabilidad del índice durante el período de contratación institucional.

El NAS, obtenido del historial electrónico, fue calculado por los enfermeros de la UTI en cuestión, diariamente, a las 17 horas. Así, se registraron para este estudio el NAS en tres momentos de la estancia del paciente en la UTI: ingreso, intermedio (día medio de hospitalización) y al salir de la unidad.

Por último, con el objetivo de garantizar la fiabilidad de los datos extraídos y reducir los sesgos de selección y de notificación de datos, estos fueron recopilados por un único investigador, utilizando un instrumento de recolección estandarizado. Además, los historiales que no contenían todas las variables notificadas tuvieron sus números de atención, utilizados para su localización, excluidos de la base de datos y, por consiguiente, del estudio.

### Análisis y Tratamiento de los Datos

Los datos se analizaron mediante el paquete estadístico R versión 4.3.0. Las variables continuas se presentaron por media, desviación estándar, mediana y cuartiles, y las categóricas, como frecuencia absoluta (n) y relativa (%). Para comprobar la influencia de las variables independientes (gravedad neurológica y nivel de dependencia) en la carga de trabajo de enfermería, se utilizó el modelo de efectos mixtos, empleando la prueba de Wald tipo III. Esta metodología permitió evaluar las mediciones de un paciente a lo largo del periodo estudiado, así como su variación. El nivel de significación adoptado fue de 0,05, en pruebas bicaudales, considerado un α del 5 %.

### Aspectos Éticos

El presente estudio siguió los principios éticos que regulan las investigaciones con seres humanos, de acuerdo con las Resoluciones n.º 466/12 y 510/16 del Consejo Nacional de Salud. Según lo recomendado por la institución, inicialmente el proyecto fue autorizado por las coordinaciones de las unidades donde se llevaría a cabo la investigación y sometido a la Central de Apoyo al Investigador (AVAP), plataforma de gestión de investigaciones del hospital donde se realizó el estudio. Posteriormente, el proyecto fue sometido al Comité de Ética en Investigación (CEP), que lo aprobó (Dictamen n.º 6.176.270 y CAAE n.º 70539323.0.0000.5461). El CEP autorizó la exención de la aplicación del Término de Consentimiento Libre y Esclarecido a los pacientes, ya que la recolección de datos se realizó de forma secundaria a través del sistema Epimed Solutions® y de las historias clínicas electrónicas.

## RESULTADOS

Al analizar el perfil de los pacientes incluidos en el presente estudio, se identificó un predominio del sexo masculino (n = 58; 62,4 %), raza blanca (n = 88; 94,6 %), estado civil casado (n = 60; 64,5 %), educación superior completa (n = 70; 75,3 %) y religión católica (n = 58; 62,3 %). La edad media fue de 76,1 ± 14,8 años, la mediana de 81 años y el índice de masa corporal (IMC) medio de 27,4 ± 4,7.

Hubo un predominio de pacientes con ACV de origen isquémico (95,7 %), ingresados en la UTI por causa clínica (94,6 %), procedentes de urgencias (82,8 %), independientes a partir de la evaluación clínica (79,6 %) y sin síntomas incapacitantes (46,2 %) según la escala de Rankin previa al ACV.

En cuanto al soporte utilizado en la UTI, el 12,9 % de la muestra requirió ventilación mecánica y el 4,3 % evolucionó a traqueotomía. El uso de vasopresores se registró en el 17,2 % de la muestra, solo el 2,1 % requirió terapia de sustitución renal y no se utilizó oxigenación por membrana extracorpórea.

La media y la mediana de la puntuación del NHISS al ingreso en urgencias fue de 8,5 ± 6,2 y 7, respectivamente, y se identificó una mayor frecuencia de déficits relacionados con la parálisis facial central (29 %), la sensibilidad (26,9 % con pérdida leve a moderada y 12,9 % con pérdida grave) y la capacidad del habla, evidenciada por disartria (25,8 % con disartria leve a moderada y 23,7 % con disartria grave) y mejor lenguaje (16,1 % con afasia grave). Los demás hallazgos relacionados con el índice de comorbilidad, la gravedad y el tiempo de estancia en la UTI y en el hospital se muestran en la Tabla 1.

**Tabla 1:** Caracterización de la muestra según el índice de comorbilidad de Charlson, gravedad y tiempo de hospitalización – São Paulo, SP, Brasil 2023.

	Media	DE	Min	C1	Mediana	C3	Max	IC min.	IC max.
NIHSS	8,5	6,2	1,0	3,0	7,0	14	24	7,3	9,8
Charlson	1,4	1,6	0,0	0,0	1,0	2,0	6,0	1,2	1,8
SAPS 3	51,3	8,3	34,0	46,0	54,0	56,0	82,0	49,6	53,0
SOFA	2,2	2,6	0,0	0,0	2,0	3,0	13,0	1,8	2,8
Tiempo de internación en UTI	5,6	6,0	1,0	2,0	3,0	7,0	40,0	4,6	7,2
Tiempo de internación	19,5	19,1	2,0	8,0	12,0	23,0	107,0	16,2	24,1
Hospitalaria									
Índice de Katz									
Previo	5,3	1,1	1,5	5,0	6,0	6,0	6,0	5,0	5,5
Admisión	3,1	1,8	0,5	1,0	3,0	4,5	6,0	2,7	3,4
Alta	3,2	2,1	0,0	2,0	3,0	4,5	6,0	2,8	3,6
NAS									
Admisión	50,8	20,3	24,8	42,7	49,4	71,3	108,7	54,1	62,3
Intermedio	56,5	17,3	31	42,3	51,6	67,8	101,1	53,2	60,2
Alta	51,7	14,7	26,6	40,8	49,3	61,5	88,5	48,9	54,8

Mín – Mínimo; Máx – Máximo; C1 – Cuartil 1; C3 – Cuartil 3; IC – Intervalo de Confianza; NIHSS – *National Institute of Health Stroke Scale*; Charlson – *Charlson Comorbidity Index*; SAPS3 – *Simplified Acute Physiology Score 3;* SOFA – *Sequential Sepsis-related Organ Failure Assessment*; UTI – Unidade de Terapia Intensiva; NAS – Nursing *Activities Score*; Katz – *Índice de Katz*.

En cuanto al Índice de Katz recopilado con los familiares, los pacientes presentaban un valor medio del estado previo de 5,3, lo que reflejaba la independencia del grupo. Tras el accidente cerebrovascular, el valor medio se redujo significativamente a 3,1, con un ligero aumento en el momento del alta de la UTI a 3,2.

En cuanto al NAS, se observó una reducción entre los momentos de medición durante la hospitalización en la UCI. En el momento del ingreso en la unidad crítica, se observaron valores más altos de NAS, con una media de 58,0 % y una mediana de 49,4 %. En la recolección intermedia se observó un cambio discreto en las puntuaciones, 56,5 % y 51,6 %, mientras que, al alta, la reducción del NAS se mantuvo, 51,7 % y 49,3 %, respectivamente.

En cuanto a la categorización de la dependencia a partir del Índice de Katz, se observó que el nivel de dependencia aumenta en todos los ítems analizados en el momento del ingreso en la UTI en comparación con el comportamiento del paciente antes del evento cerebrovascular (estado previo). Sin embargo, se observa una mejora en los porcentajes en el momento del alta, con excepción del ítem «transferencia», que presenta un aumento exponencial de la dependencia del 1,1 % en el ingreso al 30,1 % al alta de la UTI.

En relación con los ítems del NAS, se observó una frecuencia significativa de intervenciones relacionadas con los cuidados básicos, como en el ítem 4a (realización de procedimientos de higiene), con un 71 % en el ingreso en la UTI, seguido de un 68,8 % en la fase intermedia y, por último, un 76,3 % al alta. Lo mismo se aplicó al ítem 6b, necesidad de movilización y posicionamiento más de 3 veces en 24 horas o con dos profesionales de enfermería, con un 57,7 %, un 62,4 % y un 39,8 %, respectivamente, en los momentos mencionados. Otras intervenciones con hallazgos más significativos estuvieron relacionadas con la monitorización y los controles (ítem 1a); investigaciones de laboratorio, bioquímicas y microbiológicas (ítem 2); administración de medicamentos, excepto fármacos vasoactivos (ítem 3), apoyo y cuidados a los familiares (ítem 7a), tareas administrativas y gerenciales de rutina (ítem 8a) y medición cuantitativa de la diuresis (ítem 17). La Figura 1 muestra las puntuaciones de los ítems del NAS en los tres momentos medidos en la UTI.

**Figura 1:** Frecuencia de los ítems del NAS puntuados en los diferentes momentos de medición.

La Tabla 2 muestra la relación entre la puntuación del NHISS en el momento del ingreso en urgencias y la demanda de cuidados de enfermería medida por el NAS en los tres momentos (ingreso, intermedio y alta). Se observa que cada unidad del NIHSS aumenta aproximadamente 1,5 puntos en el NAS al ingreso, 1,3 en el día medio de hospitalización (intermedio) y 0,7 al alta de la UTI (p = 0,013).

**Tabla 2:** Puntuación del NHISS y valores del NAS en los diferentes momentos de medición en la UTI – São Paulo, SP, Brasil, 2023.

	Momento (NAS)	Coeficiente	DE	IC Min.	IC Max.	p-Valor
NHISS	Admisión	1,589	0,267	1,061	2,116	0,013
	Intermedio	1,335	0,267	0,808	1,863	
	Alta	0,767	0,267	0,240	1,294	

DE: desviación estándar; IC min: intervalo de confianza mínimo; IC max: intervalo de confianza máximo.

La Figura 2 muestra que cuanto mayor es el valor del NIHSS, mayor es la media marginal estimada en el NAS, independientemente del momento analizado. Además, se observa cómo cambia la demanda de cuidados requerida por el paciente en relación con su puntuación NHISS al ingreso y que los pacientes con NHISS más elevados evolucionaron con una caída significativamente más acentuada (p = 0,013) del valor del NAS durante la hospitalización, a diferencia de los ingresados en la UTI con valores más bajos en el NHISS, que presentan una menor variación de las puntuaciones del NAS registradas. Se identificó un comportamiento similar entre el índice de Katz y el NAS de los pacientes analizados en los tres momentos de la estancia en la UTI (p = 0,328).

**Figura 2:** Gráficos de la relación entre la puntuación NAS frente al NIHSS y el índice de Katz en los diferentes momentos de la medición en la UTI.

La Tabla 3 presenta el análisis de la influencia del NHISS y del Índice de Katz en la carga de trabajo de enfermería mediante el NAS medido en los tres momentos. Se observa que el NIHSS (p < 0,001) y el Índice de Katz (p = 0,002) predicen los valores del NAS de forma independiente y no hay relación con el momento en que se mide la carga de trabajo de enfermería. Así, los pacientes más dependientes para las AVD (valores más bajos del Índice de Katz) y aquellos con mayor gravedad neurológica observada por el NIHSS tendrán puntuaciones NAS más altas, independientemente del momento de la medición (ingreso, intermedio o alta).

**Tabla 3:** Influencia del Índice de Katz y del NHISS en la carga de trabajo medida por el NAS en la UTI en diferentes momentos – São Paulo, SP, Brasil, 2023.

	X^2^	p-Valor (>X^2^)
(Intercepto)	218,862	<0,001
Momento de la medida	0,385	0,535
NIHSS	13,615	<0,001
Katz	10,002	0,002
Momento: NIHSS	3,366	0,067
Momento: Katz	0,957	0,328

X^2^ – chi-cuadrado de Pearson; NIHSS – *National Institute of Health Stroke Scale*; Katz – *Índice de Katz.*

Por último, la mayoría de los pacientes fueron dados de alta de la UTI (94,6 %) y sobrevivieron (91,4 %) durante la hospitalización.

## DISCUSIÓN

El presente estudio evidenció que el NIHSS y el índice de Katz influyen en los valores del NAS de forma independiente, sin relación con el momento de la medición de la carga de trabajo de enfermería en la UTI (admisión, estado intermedio o alta).

La muestra estaba compuesta predominantemente por hombres y ancianos. En este sentido, se sabe que las enfermedades crónicas asociadas a factores como la edad avanzada y la hospitalización en la UTI afectan negativamente a la independencia funcional de los individuos, de modo que, a medida que aumenta la incidencia de enfermedades, mayor es el riesgo de incapacitación en las actividades básicas de la vida^(15)^.

Por lo tanto, cabe destacar que los eventos cerebrovasculares tienen una asociación directa con el grado de dependencia de las personas mayores^(16)^. Así, se justifica el hecho de que los pacientes presenten, antes del ictus, un mayor grado de independencia funcional con una posterior disminución de la puntuación en el índice de Katz, volviéndose más dependientes después del evento cerebrovascular.

Además, el conjunto de déficits neurológicos que presentan los pacientes como consecuencia de alteraciones vasculares está íntimamente relacionado con su gravedad. Los resultados presentes en la literatura han demostrado la existencia de una relación entre los síntomas leves de ACV, señalados por la escala NHISS, y la expansión o el área de la lesión neurológica^(16)^. Teniendo en cuenta que las puntuaciones entre 8 y 17 puntos caracterizan una gravedad neurológica moderada, el presente estudio evidenció una puntuación media de 8,5 en la NHISS asociada a una mayor frecuencia de déficits relacionados con la parálisis facial central, la sensibilidad y la capacidad del habla, según lo señalado por los índices de disartria y mejor lenguaje, además de alteraciones en la fuerza motora.

Las alteraciones relacionadas con el lenguaje, la negligencia, los déficits visuales y las actividades motoras y sensoriales, generalmente presentes en casos considerados graves, suelen estar relacionadas con resultados menos favorables y, en consecuencia, con un mayor grado de dependencia tras el evento neurológico^(17)^. De este modo, la presencia de tales déficits en la muestra en cuestión puede estar relacionada con cambios en los grados de dependencia para la ejecución de las AVD.

Además, se sabe que el principal motivo de los ingresos de personas mayores en la UTI es la agudización de enfermedades crónicas, principalmente de origen vascular, como ocurre en el ACV^(17,18)^. La asistencia de enfermería prestada a esta población está asociada al aumento de la carga de trabajo de enfermería^(19)^, como lo evidencian las puntuaciones medias de la NAS al ingreso en la UTI (58,0 %), en el momento intermedio (56,5 %) y al alta (51,7 %). Se encontraron resultados similares en un estudio realizado por Lucchini et al.^(19)^, que registró una NAS media de 59,33 en UTIs de Italia, así como en un estudio brasileño que analizó a ancianos que necesitaron cuidados intensivos^(20)^.

Ante esto, como consecuencia de los cambios fisiológicos causados por el propio proceso de envejecimiento, a menudo asociados a determinados grados de fragilidad, este público se vuelve más vulnerable, hecho que a menudo se ve potenciado por las condiciones patológicas que lo rodean, como, por ejemplo, las incapacidades causadas por el ictus^(21,22)^. En lo que respecta al grupo en cuestión, se observó un predominio de cuidados relacionados con actividades básicas, como la movilización/posicionamiento y los procedimientos de higiene, incluido el baño en la cama, presentes durante toda la estancia en la UTI. Por lo general, las actividades relacionadas con la higiene y la movilización están asociadas a una pérdida progresiva de la autonomía para realizar actividades básicas e instrumentales, acentuada por los diversos grados de déficit provocados por el ictus^(8)^.

Otras intervenciones más frecuentes en todos los momentos de la hospitalización se relacionaban con la necesidad de monitorización y signos vitales (ítem 1), investigación microbiológica (ítem 2), medicación, excepto fármacos vasoactivos (ítem 3), y apoyo y cuidados a los familiares (ítem 7). Estos hallazgos se justifican una vez más por el rango etario predominante en el grupo, que requiere una mayor monitorización continua, como consecuencia de los cambios sufridos por el organismo, afectados principalmente por las condiciones de salud que impactan directamente en el uso de medicamentos de uso continuo, con el fin de controlar dichas condiciones, y que generalmente dan lugar a la llamada polifarmacia^(23,24)^.

Teniendo en cuenta las propiedades psicométricas del Índice de Katz y sus versiones, se ha identificado que es un instrumento válido y fiable para determinar y evaluar la capacidad para realizar AVD y la mortalidad de pacientes post-ictus^(8,25)^. En este sentido, el estudio en cuestión demostró la relación entre la gravedad neurológica y la capacidad para realizar las AVD, en comparación con el grado funcional previo. Con una interacción representada por coeficientes negativos, se observó que, tras el evento cerebrovascular, hubo una influencia del ictus sobre la capacidad funcional en la siguiente proporción: se redujo en 0,299 el índice de Katz al ingreso y en 0,332 el resultado final en relación con su capacidad previa, aumentando el nivel de dependencia de la muestra.

Además, las pruebas apuntan a la influencia directa del ACV como agente incapacitante para la funcionalidad y la independencia física de un individuo, lo que demuestra una relación directa y aceptable con el índice de Katz, considerado un excelente predictor de incapacidades tras el evento, especialmente al asociar edades más avanzadas con mayores grados de incapacidad^(26)^. En este sentido, el estudio en cuestión muestra una interacción directa entre la aparición del ACV y la alteración de su capacidad funcional. Dada la frecuencia de las interacciones entre la gravedad de la enfermedad y la carga de trabajo de enfermería, se sabe que la escala NHISS actúa como un indicador de gravedad neurológica, generalmente relacionado con la extensión y la gravedad del evento cerebrovascular. El presente estudio evidenció que los pacientes que presentaban mayor gravedad tenían inicialmente puntuaciones de mayor demanda asistencial, así como una caída más acentuada de este índice durante el proceso de ingreso en la UTI. Los hallazgos presentes en la literatura demostraron la existencia de esta relación de manera similar, al evidenciar en una UTI neurológica la presencia de mayores puntuaciones NAS asociadas a pacientes que presentan mayor gravedad según el SAPSII^(13)^.

De este modo, se sabe que la gravedad de la enfermedad influye en los cuidados prestados al paciente y que, junto con ello, su dependencia física afectará a la realización de determinadas actividades. Sin embargo, la gravedad de la enfermedad no debe ser el único factor a tener en cuenta para determinar los cuidados de enfermería prestados, ya que, dada la pluralidad que presentan los pacientes, deben considerarse otros factores, como la dependencia funcional y el nivel de conciencia^(8,19,27)^. En este sentido, se evidenció una interacción independiente entre las variables citadas (NHISS e Índice de Katz) con el NAS, lo que permite inferir que tanto la gravedad neurológica como el grado de incapacitación influirán en la demanda de cuidados de enfermería requeridos por los pacientes en la UTI, independientemente del momento de la medición. Sin embargo, el estudio presenta las siguientes limitaciones: la presencia de una muestra perteneciente a una única institución sanitaria, con una población con acceso a asistencia sanitaria complementaria y un mayor seguimiento clínico a largo plazo, lo que puede limitar su validez externa.

Además, cabe destacar la ausencia de un análisis por separado del grupo estudiado por tipo de evento (isquémico o hemorrágico), ya que los datos se presentan de forma unificada.

Por otro lado, cabe destacar la innovación de esta investigación, ya que no existen trabajos en esta línea, es decir, que señalen la relación entre el trastorno neurológico abordado y la demanda de cuidados de enfermería en terapia intensiva. Junto a esto, destaca la buena estructura del diseño establecido para el análisis de los datos, lo que aporta solidez a los hallazgos presentes en este estudio.

Por último, considerando que la carga de trabajo de enfermería interfiere directamente en la calidad de la atención^(28)^, los resultados aquí presentados tienen un gran potencial para contribuir a la práctica clínica y a la investigación, ya que destacan, de forma inédita, la asistencia de enfermería intensiva prestada a pacientes con diagnóstico clínico de ACV. De este modo, las evidencias relativas a la carga de trabajo requerida por los pacientes post-ACV ingresados en la UTI contribuyen al fortalecimiento de la gestión en salud al identificar aspectos que pueden ayudar a dimensionar el equipo de enfermería y la distribución diaria de los pacientes por profesional en función de la gravedad neurológica, el grado de dependencia y otras condiciones pertinentes para garantizar la seguridad de la asistencia intensiva prestada.

Aunque este estudio se ha llevado a cabo con una muestra superior a la estimada por el cálculo estadístico, se cree que la realización de nuevas investigaciones sobre el NAS en pacientes con ACV, especialmente multicéntricas y en otros contextos sanitarios, como instituciones públicas y unidades no críticas, puede fortalecer y fundamentar la práctica asistencial de esta población mediante la consolidación de más evidencias científicas.

## CONCLUSIÓN

En vista de lo anterior, se concluye que el grado de gravedad neurológica presentado por la puntuación NHISS y la dependencia funcional según el índice de Katz presentados por los pacientes post-ACV influyen significativamente en la carga de trabajo de enfermería medida por el NAS, independientemente del momento de su medición durante la hospitalización en la UTI.

## Data Availability

El conjunto completo de datos que respalda los hallazgos de este estudio está disponible previa solicitud al autor correspondiente, Felipe da Cruz Lima. El conjunto de datos no está disponible públicamente debido a que los resultados de la investigación contienen información que compromete la privacidad de los participantes.
